# Discriminative Learning Approach Based on Flexible Mixture Model for Medical Data Categorization and Recognition

**DOI:** 10.3390/s21072450

**Published:** 2021-04-02

**Authors:** Fahd Alharithi, Ahmed Almulihi, Sami Bourouis, Roobaea Alroobaea, Nizar Bouguila

**Affiliations:** 1 College of Computers and Information Technology, Taif University, Taif, P.O. Box 11099, Taif 21944, Saudi Arabia; a.almulihi@tu.edu.sa (A.A.); s.bourouis@tu.edu.sa (S.B.); r.robai@tu.edu.sa (R.A.); 2 The Concordia Institute for Information Systems Engineering (CIISE), Concordia University, Montreal, QC H3G 1T7, Canada; nizar.bouguila@concordia.ca

**Keywords:** shifted-scaled Dirichlet distribution, mixture model, SVM kernels, data categorization and recognition, medical image analysis

## Abstract

In this paper, we propose a novel hybrid discriminative learning approach based on shifted-scaled Dirichlet mixture model (SSDMM) and Support Vector Machines (SVMs) to address some challenging problems of medical data categorization and recognition. The main goal is to capture accurately the intrinsic nature of biomedical images by considering the desirable properties of both generative and discriminative models. To achieve this objective, we propose to derive new data-based SVM kernels generated from the developed mixture model SSDMM. The proposed approach includes the following steps: the extraction of robust local descriptors, the learning of the developed mixture model via the expectation–maximization (EM) algorithm, and finally the building of three SVM kernels for data categorization and classification. The potential of the implemented framework is illustrated through two challenging problems that concern the categorization of retinal images into normal or diabetic cases and the recognition of lung diseases in chest X-rays (CXR) images. The obtained results demonstrate the merits of our hybrid approach as compared to other methods.

## 1. Introduction

Unsupervised data categorization and recognition are widely used tools in statistical data analysis and are progressively being applied to complex datasets allowing the discovery of similar statistical patterns. They have been applied in a diversity of applications, ranging from the fields of image processing, data mining, to those of biomedicine, security and social media. Nowadays, the trend of data mining applications in healthcare is remarkable as there are huge datasets in this sector that require a deep analysis by effective model-based techniques derived from artificial intelligence and machine learning areas. Large scale artificial intelligence and machine learning tools are increasingly successful in image-based diagnosis and have been employed for medical decision making [1,2,3,4,5] and other complex problems like scene and web pages categorization [6,7,8], retinal images classification [9] and action recognition [10]. The manual processing of these tasks is difficult, tedious and time consuming and so it is important to move to automatic methods, which are able to learn models from labeled and non-labeled data and allow faster and more accurate decisions. Most of the model-based techniques applied to classification and recognition problems are approached through finite mixture models and the commonly used mixtures are based on Gaussian assumption [11]. Mixture models are designed as well principled statistical models and have the advantage of using different distributions in order to describe its components. They are used for effectively modeling visual features thanks to their capability of describing multidimensional distributions and heterogeneous data in a finite number of classes [11,12,13]. They focus on modeling a given distribution by weighted sum (i.e., a mixture) of several basic distributions (combination of two or more probability density functions).

It is noted that while the data follows a non-Gaussian distribution in nature, the Gaussian model can give a weak performance. Noticing this fact, various mixture models have been proposed in the literature and some distinguished ones are based on the generalized Gaussian [14], Dirichlet and generalized Dirichlet [15], Beta-Liouville [15], t-student distributions, etc. For instance, the Dirichlet mixture and its extensions (like generalized Dirichlet) have been successfully employed and can often outperform the Gaussian model for data clustering, categorization and action recognition [9,16,17,18,18]. In this context, the work in this paper is based on recent research outcomes that have shown the importance of some specific distributions for complex visual vectors modeling based on Dirichlet and scaled Dirichlet mixture distributions [16,19]. In particular, some recent studies have shown that the derived model, the called shifted-scaled Dirichlet mixture model (SSDMM), can be applied successfully for a variety of applications [20,21]. SSDMM is presented as a powerful generalization of both Dirichlet and scaled Dirichlet where the term shifted, here, means a perturbation in the simplex.

It is noteworthy that several generative probabilistic models have a lot of benefits in terms of their capability to categorize similar data and analyze complex data, but when the data are heavily corrupted due to noise and outliers, these models sometimes fail. To cope with these disadvantages, it is possible to envisage applying discriminative classifiers, in particular Support Vector Machines (SVM), instead. However, most of the conventional classifiers do not take into account the nature of input data and therefore fail to reach very good results. Consequently, it is better to think of a hybrid approach that considers both the benefits of generative and discriminative models in order to reach better performance. This objective can be achieved for instance by generating powerful mixture-based probabilistic SVM kernels.

### Motivations and Contributions

In this paper, we propose to investigate recent research outcomes that have shown the importance of the shifted-scaled Dirichlet mixture model (SSDMM) and then we go a step further by developing a discriminative learning approach. First of all, we address in this work an important step in mixture modeling problems, which is the model complexity problem, and we propose to solve it in order to avoid the over-fitting issue. Indeed, in order to determine the optimal statistical mixture model with less complexity we investigate an effective criterion named Minimum Message Length (*MML*) [22]. This criterion has the advantage of providing better generalization capabilities. Our second contribution is to develop a family of SVM kernels generated from the finite mixture of SSDMM and its approximation. In particular, we propose to derive some kernels on the basis of probabilistic distances in order to tackle the problem of data classification using SVM. It is noted that classical SVM kernel functions are used to compute the similarity between vectors and not between mixture of distributions, which limit their use in terms of capturing the intrinsic properties of the data to classify. In this work, we propose to address certain practical shortcomings of standard kernels by taking into account the prior knowledge of the problem at hand via mixture models. Thus, we propose to derive new kernels for SVM from generative models, which are able to take into account the complexity of data, in order to enhance its modeling and categorization capabilities. To the best of our knowledge, this is the first time a hybrid approach based on SSDMM and some derived probabilistic SVM kernels, such as Fisher kernel, Kulback–Leibler kernel, and Bhattacharyya-based kernel [23,24,25], has been developed. As a result, we expect an improvement in terms of image modeling and categorization, which can be achieved by our hybrid framework.

This paper is structured as follows. A brief introduction that describes our motivation is given in Section 1. The generative model based on the shifted-scaled Dirichlet mixture and its learning algorithm is presented in Section 2. The discriminative hybrid learning approach is developed in Section 3. In Section 4, we summarize our complete algorithm. Section 5 shows the merits of our work through extensive experiments related to two important applications namely lung disease recognition and retinopathy detection. Finally, Section 6 summarizes this manuscript and emphasizes some potential future works.

## 2. Finite Shifted-Scaled Dirichlet Mixture Model

As a part of our research, we will demonstrate that the shifted scaled Dirichlet distribution can be applied with conjunction of discriminative classifiers to model and discriminate complex biomedical multidimensional data. Let us define $\vec{Y}=\left(Y_{1},\ldots ,Y_{D}\right)$ a random vector. We say that $\vec{Y}$ follows a shifted scaled Dirichlet distribution with parameters $\theta =(\vec{\alpha },\vec{\beta },b)$ such that $\vec{\alpha }=\left(\alpha_{1},\ldots ,\alpha_{D}\right)\in R_{+}^{D}$, $\vec{\beta }=\left(\beta_{1},\ldots ,\beta_{D}\right)\in S^{D}$ and $b\in R_{+}$ if its density function is defined as [26]:

(1) $$p\left(\vec{Y}|\theta \right)=\frac{\Gamma (\alpha_{+})}{\prod_{i=1}^{D}\Gamma \left(\alpha_{i}\right)}\frac{1}{b^{D-1}}\frac{\prod_{i=1}^{D}\beta_{i}^{-(\alpha_{i}/b)}y_{i}^{(\alpha_{i}/b)-1}}{{\left(\sum_{i=1}^{D}{\left(y_{i}/\beta_{i}\right)}^{\left(1/b\right)}\right)}^{\alpha_{+}}}$$

where $\vec{\beta }$ denotes a location parameter. $\alpha_{+}=\sum_{d=1}^{D}\alpha_{d}$. Γ(.) is the Gamma function. The shifted-scaled Dirichlet distribution has 2D parameters. If the parameter a=1, then this model is reduced to a scaled Dirichlet model.

A mixture of *K*-components of SSDMM distributions can be written as:

(2) $$p\left(\vec{Y}|\Theta \right)=\sum_{k=1}^{K}\pi_{k}p\left(\vec{Y}|\theta_{k}\right)$$

where Θ is the set of all model’s parameters: $\Theta =(\pi_{k},\theta_{k})$. Here, we denote $\pi_{k}$, k=1,…,K by the mixing proportion that must satisfy the unity condition (are positive and their sum is equal to one). Let Y be a set of vectors, $Y=\{{\vec{Y}}_{1},{\vec{Y}}_{2},\ldots ,{\vec{Y}}_{N}\}$, such that each vector is a realization from a *K*-component mixture. Each sample vector ${\vec{Y}}_{n}=\left(y_{n1},\ldots ,y_{nD}\right)$ is D-dimensional. The log-likelihood function of the complete dataset is as:

(3) $$L\left(Y|\Theta \right)=\prod_{n=1}^{N}\sum_{k=1}^{K}\pi_{k}p\left({\vec{Y}}_{n}|\theta_{k}\right)$$

where $p({\vec{Y}}_{n}|\theta_{k})$ is the shifted-scaled Dirichlet probability with parameter $\theta_{k}=\left({\vec{\alpha }}_{k},{\vec{\beta }}_{k},b_{k}\right)$.

In order to estimate the parameters of the mixture Θ, we consider here a widely applied technique, which is the Maximum Likelihood Estimate (MLE) via the well known algorithm expectation–maximization (EM) [27]. This algorithm is able to provide several estimates $\left\{\Theta^{t},t=0,1,2\ldots \right\}$ by alternating between the following two steps (E-step) and (M-step) until convergence based on certain criteria:

1. **Initialization-step:** Apply K-means algorithm to initialize the parameters of the mixture.
2. **E-step:** Calculate the posterior probability ${\hat{Z}}_{ij}$ as:
  (4) $${\hat{Z}}_{ij}=\frac{p\left(\vec{Y_{i}}|\theta_{j}\right)\pi_{j}}{\sum_{l=1}^{M}p\left(\vec{Y_{i}}|\theta_{l}\right)\pi_{l}}$$
3. **M-step:** Update the model’s parameter by maximizing the log-likelihood function as:
  (5) $$\begin{array}{cc} \hat{\Theta }= & \arg\max_{\Theta }L\left(\Theta ,Z,Y\right)\\ = & \arg\max_{\Theta }\prod_{i=1}^{N}\sum_{j=1}^{M}Z_{ij}\log\left(p\left(\vec{Y_{i}}|\theta_{j}\right)\pi_{j}\right) \end{array}$$
  where the membership $Z=\{{\vec{Z}}_{1},\ldots ,{\vec{Z}}_{N}\}$, ${\vec{Z}}_{i}=\left(Z_{i1},\ldots ,Z_{iM}\right)$ with:
  (6) $$Z_{ij}=\left\{\begin{array}{cc} 1 & \mathrm{if}\vec{Y_{i}}\in \mathrm{component}j\\ 0 & \mathrm{otherwise} \end{array}\right.$$

In the current study, the implemented learning statistical model takes into account the problem of model complexity. Indeed, determining the optimal model *M* is an important step in mixture modeling problems, which is able to provide better generalization capabilities. To evaluate statistical models, we proceed with a criterion derived from information theory named “Minimum Message Length criterion (*MML*)” as it was successfully applied previously in [22]. It is noted that *MML* has the advantage to accurately estimate the model complexity and returns the optimal number of components in the mixture by minimizing this function:

(7) $$\begin{array}{ccc} MML(\Theta ,Y) & ≃ & -log\left(p\left(\Theta \right)\right)-L\left(\Theta ,Y\right)+\frac{1}{2}log\left|F\left(\Theta \right)\right|+\frac{N_{p}}{2}+\frac{N_{p}}{2}log\left(K_{N_{p}}\right)\\ \; & ≃ & -log\left(p\left(\Theta \right)\right)-L\left(\Theta ,Y\right)+\frac{1}{2}log\left|F\left(\Theta \right)\right|+\frac{N_{p}}{2}+\frac{N_{p}}{2}log\left(12\right) \end{array}$$

where p(Θ) is a prior probability for the model, |F(Θ)| is the determinant of the Fisher information matrix, and $N_{p}$ is the number of parameters where it is in our case equal to K(2D+1)−1. Here, the parameter $K_{N_{p}}$ is named as the optimal quantization lattice constant for $R^{Np}$. It is noted that $K_{1}=1/12≃0.083$, for $N_{p}=1$. Moreover, as $N_{p}$ increases, $K_{N_{p}}$ tends to an asymptotic value equal to $\frac{1}{2\pi e}≃0.05855$, which can be approximated by $\frac{1}{12}$ [13,22,28] (since $K_{N_{p}}$ does not vary much).

In the following, we determine an appropriate a prior distribution, p(Θ), and we conduct an expression for |F(Θ)|. As there is no prior knowledge about the model’s parameters (i.e., all mixture’ components are independent), thus we proceed by modeling these parameters as follows:

(8) p(Θ)=p(α)p(β)p(b)p(π)

It is noted that both the location parameter β is defined on the simplex such that $\sum_{d=1}^{D}\beta_{d}=1$. In the same way, the mixing weight π, it is defined on the simplex and we have $\sum_{j=1}^{K}\pi_{j}=1$. For such reason, it is a natural choice to choose a Dirichlet prior with different hyperparameters Dir(π|u) and Dir(β|v) for both location and mixing parameters, respectively. These priors are defined as:

(9) $$p\left(\pi \right)=Dir\left(\pi |u\right)=\frac{\Gamma (\sum_{i=1}^{K}u_{i})}{\prod_{i=1}^{K}\Gamma \left(u_{i}\right)}\prod_{i=1}^{K}\pi^{u_{i}-1}$$

(10) $$p\left(\beta \right)=Dir\left(\beta |v\right)=\frac{\Gamma (\sum_{d=1}^{D}v_{d})}{\prod_{d=1}^{D}\Gamma \left(v_{d}\right)}\prod_{d=1}^{D}\beta^{v_{d}-1}$$

For the scale parameter, we determine it experimentally and the good choice for this scalar is found equal $p\left(b\right)=\frac{1}{10}$. Finally, given there is no knowledge regarding the shape parameter α, we suppose that all $\alpha_{j}$ are independent. Thus, we proceed by taking a simple uniform prior. According to Ockham’s razor, it has been previously proven that this choice is able to give stable results [29]. We have:

(11) $$p\left(\alpha \right)=\prod_{j=1}^{K}p\left(\alpha_{j}\right)=\prod_{j=1}^{K}\prod_{d=1}^{D}p\left(\alpha_{jd}\right)$$

where $p\left(\alpha_{jd}\right)=e^{6}\frac{\alpha_{jd}}{\left|\right|\alpha_{jd}\left|\right|}$. Here $\alpha_{jd}$ denotes the estimated shape vector and $\left|\right|\alpha_{jd}\left|\right|$ corresponds to its norm.

Now, regarding the Fisher information matrix, it is noted that calculating this quantity for the case of mixture models is very complex since there is no analytical form. For such reason, we approximate this quantity by adopting the determinant of Fisher Information matrix as

(12) $$\left|F\left(\Theta \right)\right|=\left|F\left(\pi \right)\right|\prod_{j=1}^{K}|F\left(\theta_{j}\right)|=|F\left(\pi \right)|\prod_{j=1}^{K}|F\left(\alpha_{j}\right)||F\left(\beta_{j}\right)||F\left(b_{j}\right)|$$

where $\left|F(\theta_{j})\right|$ is the determinant of the Fisher information of the parameters $\theta_{j}=\left(\alpha_{j},\beta_{j},b_{j}\right)$ to be estimated. The Fisher information related to the mixing weights is given as:

(13) $$\left|F\left(\pi \right)\right|=\frac{N}{\prod_{j=1}^{K}\pi_{j}}$$

For $\left|F(\theta_{j})\right|$, it is calculated by taking the determinant of the Hessian matrix of the negative log-likelihood function (i.e., second derivative of the log-likelihood).

## 3. Discriminative Learning Approach Based on SSDMM

To deal with the disadvantages of generative models, one could apply instead discriminative classifiers to improve expected results for data categorization and or recognition. Actually, many classifiers such as SVM show great potential compared to generative models for several applications [30,31]. However, in most applications, the conventional SVMs kernels (i.e., linear, polynomial, RBF) [30] are not able to consider the nature of the data and it was noted that choosing conventional SVM kernels was not the right choice [14]. This disadvantage limits their performance. This problem has been well solved, for example, by combining a discriminative classifier (such as SVM) with a generative learning model into the same framework [9]. Indeed, building new SVM kernels directly from data, using for instance information divergence or Fisher score [23,24] between distributions, may lead to better performance. Thus, a hybrid approach could be a good choice, which is designed as a compromise between generative and discriminative ones. In this current work, we suggest to construct new data-based probabilistic classifiers through the shifted scaled Dirichlet mixture model (SSDMM). It is noted that the resulting hybrid model has the advantage of merging the strengths of both generative and discriminative models and thus of increasing performance given that we will take into account the intrinsic structure of input data (since we measure the similarity between input vectors). In this section, we focus on generating three probabilistic kernels named as Fisher kernel, Kulback–Leibler kernel, and Bhattacharyya-based kernel [23,24,25]. These kernels are developed as follows.

**The Fisher Kernel (FK):** The key intuition behind the Fisher kernel is to exploit the geometric structure on the statistical manifold and it is defined in the gradient log-likelihood space [23], which is obtained by calculating the gradient of sample log-likelihood as regards to the model parameters. Therefore, similar mixtures involve similar log-likelihood gradients. The similarity between SSDMM mixtures (*Y* and $Y^{\prime }$) based on the Fisher kernel is defined as:

(14) $$\mathrm{FK}\left(Y,Y^{\prime }\right)=U_{Y}^{tr}\left(\Theta \right)I^{-1}\left(\Theta \right)U_{Y^{\prime }}\left(\Theta^{\prime }\right)$$

The derivatives of the log-likelihood (i.e., the gradient of log(p(Y|Θ))) with respect to parameters Θ are expressed as:

(15) $$U_{Y}\left(\Theta \right)=\nabla \log\left(p\left(Y|\Theta \right)\right)=\frac{\partial \log(p(Y|\Theta ))}{\partial \Theta }$$

To solve the previous equation, we need to compute the gradient with respect to parameters $\pi_{j}$ and $\alpha_{j},j=1,\ldots ,K,$. The resulting derivative equations are given as:

(16) $$\frac{\partial \log(p(Y|\Theta ))}{\partial \pi_{j}}=\sum_{i=1}^{N}\left[\frac{{\hat{Z}}_{ij}}{\pi_{j}}-\frac{{\hat{Z}}_{i1}}{\pi_{1}}\right]$$

(17) $$\frac{\partial \log(p(Y|\Theta ))}{\partial \alpha_{j}}=\sum_{i=1}^{N}{\hat{Z}}_{ij}\left(\psi \left(\alpha_{+}\right)-\psi \left(\alpha_{j}\right)+\frac{log\left(Y_{n}\right)-log\left(\beta_{j}\right)}{b_{j}}-log\left(\sum_{d=1}^{D}{\left(\frac{Y_{nd}}{\beta_{jd}}\right)}^{\frac{1}{b_{j}}}\right)\right)$$

where ψ is the digamma function.

**The Symmetrized Kullback–Leibler Kernel (SKK):** We propose here to compute the symmetrized Kullback–Leibler distance (SKK) [24] to measure the degree of similarity between two mixture models. This symmetrized distance has the advantage of offering a more balanced measurement than the asymmetrized one and makes it more appropriate for the classification task. It has been shown that, in speaker recognition, the Gaussian mixture model (GMM) and SVM with Kullback–Leibler (KL) kernel [32] can give good performance. It is also noted that, in a conventional hybrid system, the SKK measure has often been applied to calculate the distance between two distributions and not between mixtures. Subsequently, we propose here to develop new generative SVM-SKK kernels based on SSDMM mixture models. The dissimilarity (SKK) between two mixtures $p_{1}$ and $p_{2}$ is given as:

(18) $$\begin{array}{c} \mathrm{SKK}(p_{1}\left(\vec{Y}|\Theta_{1}\right),p_{2}\left(\vec{Y^{\prime }}|\Theta_{2}\right))=\\ \mathrm{KL}(p_{1}\left(\vec{Y}|\Theta_{1}\right),p_{2}\left(\vec{Y^{\prime }}|\Theta_{2}\right))+\\ \mathrm{KL}(p_{2}\left(\vec{Y^{\prime }}|\Theta_{2}\right),p_{1}\left(\vec{Y}|\Theta_{1}\right)) \end{array}$$

(19) $$\mathrm{KL}\left(p_{1}\left(\vec{Y}|\Theta_{1}\right),p_{2}\left(\vec{Y^{\prime }}|\Theta_{2}\right)\right)=e^{-BF(p_{1}\left(\vec{Y}|\Theta_{1}\right),p_{2}\left(\vec{Y^{\prime }}|\Theta_{2}\right))}$$

where *B* is a real positive factor used for computational stability purpose, and

$$\begin{array}{cc} \; & F(p_{1}\left(\vec{Y}|\Theta_{1}\right),p_{2}\left(\vec{Y^{\prime }}|\Theta_{2}\right))=\\ \; & \int_{\omega }p_{1}\left(\vec{Y}|\Theta_{1}\right)\log\frac{p_{1}\left(\vec{Y}|\Theta_{1}\right)}{p_{2}\left(\vec{Y^{\prime }}|\Theta_{2}\right)}+p_{2}\left(\vec{Y^{\prime }}|\Theta_{2}\right)\log\frac{p_{2}\left(\vec{Y^{\prime }}|\Theta_{2}\right)}{p_{1}\left(\vec{Y}|\Theta_{1}\right)} \end{array}$$

Unfortunately, a closed form expression does not exist in the case of the SSDMM mixture. Therefore, we propose the use of a sampling approach based on the Monte Carlo numerical approximation method [33]:

(20) $$\mathrm{SKK}\left(p_{1}\left(\vec{Y}|\Theta_{1}\right),p_{2}\left(\vec{Y^{\prime }}|\Theta_{2}\right)\right)\approx \frac{1}{L}\sum_{i=1}^{L}\log\frac{p_{1}\left({\vec{Y}}_{i}|\Theta_{1}\right)}{p_{2}\left({\vec{Y^{\prime }}}_{i}|\Theta_{2}\right)}$$

**The Bhattacharyya kernel (BK):** In this study, we exploit another kernel distance derived from the family of probability product kernels called the Bhattacharyya kernel [33] (known also as Bhattacharyya’s symmetry measure of affinity between distributions). Within this kernel we can inject the domain knowledge and invariance of the SSDMM generative model to the SVM classifier. Here, a general inner product is evaluated as the integral of the product of pairs of distributions (or mixtures) and defined as:

(21) $$\begin{array}{ccc} {\mathrm{BK}}_{\frac{1}{2}}\left({\vec{Y}}_{1},{\vec{Y}}_{2}\right) & = & \int_{0}^{\infty }p{\left(\vec{Y}|\Theta_{1}\right)}^{1/2}q{\left(\vec{Y}|\Theta_{2}\right)}^{1/2}d\vec{Y} \end{array}$$

In the absence of a closed form for the generative SSDMM mixture, it is possible to approximate BK using the Monte Carlo simulation method [33].

(22) $$\begin{array}{ccc} {\mathrm{BK}}_{\frac{1}{2}}\left({\vec{Y}}_{1},{\vec{Y}}_{2}\right) & \approx & \frac{\beta }{N_{1}}\sum_{i=1}^{N_{1}}\frac{p^{1/2}\left({\vec{Y}}_{i}|\Theta_{1}\right)}{Z_{1}}p^{1/2}\left({\vec{Y}}_{i}|\Theta_{1}\right)\\ & + & \frac{1-\beta }{N_{2}}\sum_{i=1}^{N_{2}}\frac{q^{1/2}\left({\vec{Y}}_{i}|\Theta_{2}\right)}{Z_{2}}q^{1/2}\left({\vec{Y}}_{i}|\Theta_{2}\right) \end{array}$$

where β∈[0,1] and the normalized factors $Z_{1}$, $Z_{2}$ are used for the densities *p* and *q*.

## 4. Complete Algorithm

**Figure 1:**
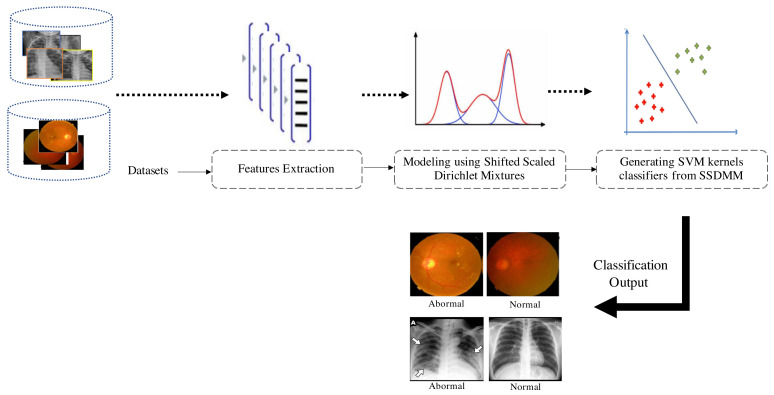
Steps of the developed approach. After extracting local features from each image, we move to the modeling step using the flexible mixture model (SSDMM). Finally, we feed the Support Vector Machines (SVM) Kernel matrices, which are built to classify images as normal or abnormal.

The proposed hybrid approach includes different steps that must be taken in order to achieve optimum performance. In this work, a first preprocessing step is performed to extract robust visual features from each image in the dataset. Indeed, image description is one of the crucial steps in many medical image processes, and extracting informative patterns (color, shape, or/and texture) help further steps such as image interpretation and classification. For instance, many image modalities (like X-rays) are difficult to interpret directly by radiologists; thus, it is important to extract relevant details and patterns for better image understanding and decisions. Thus, each image in the dataset was represented as a bag of feature vectors (i.e., the Speeded Up Robust Features (SURF) or the Haralick texture features). The second step (i.e., the generative stage of the hybrid framework) is performed by fitting the generative SSDMM model to the feature vectors extracted from images. Consequently, each image in our dataset is modeled by a finite mixture model of distributions SSDMM. We start by initializing the mixing weight π and model parameters Θ with the conventional K-Means algorithm. After that, the statistical model is learned using the maximum likelihood principle and the parameters are estimated through the expectation–maximization (EM). The last step (the discriminative stage of the hybrid framework) is dedicated to compute different probabilistic distances between each of these mixture models that give us kernel matrices to feed the SVM classifier. In particular, we focus on deriving some effective measures based on Fisher, Kullback–Leibler and Bhattacharyya kernels for the mixtures of shifted scaled Dirichlet distributions. The goal is to calculate the dissimilarity between mixtures that generates the different kernels (matrices). The resulting matrices are fed to the SVM classifier to classify images (vectors). Finally, the implemented algorithm will be trained with the computed kernel matrices using the one-versus-all training approach and perform classification results using 10-fold cross-validation. The implemented hybrid framework is summarized in Algorithm 1 and the different steps are illustrated in Figure 1.

 **Algorithm 1:** Discriminative learning approach based on SSDMM. 
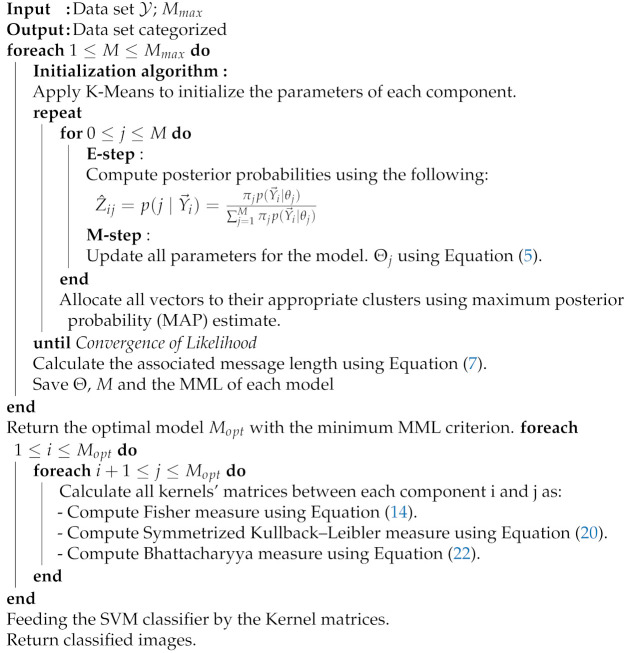


## 5. Experimental Results

The purpose of this section is to validate the proposed approach. We have considered some real medical databases for performance evaluation.

### 5.1. Lung Disease Recognition

Lung disease is one of the most common diseases worldwide. It may include pneumonia, tuberculosis, asthma, and chronic obstructive pulmonary disease. Pneumonia is one of the most serious respiratory diseases, which can be due to the causative virus of COVID-19 [34,35] or may be caused by bacterial or viral infection in the lungs [36]. In developing countries, the danger of pneumonia is enormous as thousands of people face air pollution and poverty. In December 2019, a new disease, named the COVID-19 pandemic, appeared in China and was rapidly spread worldwide [34]. This virus is a highly contagious disease. It primarily occurs by a severe acute respiratory syndrome [37]. The World Health Organization (WHO) has declared the novel virus outbreak a global pandemic of our time. Nowadays, the study of COVID-19 in particular and pneumonia in general has attracted a lot of attention due to its strong spread and quite high mortality. Early diagnosis of this disease is of great importance as it will significantly reduce its death rate. Recently, chest X-ray (CXR) radiography diagnosis is assumed to be one of the effective methods for the detection of pneumonia since it is less sensitive than CT-scan in diagnosis of lung diseases [38]. The rapid and accurate diagnosis of these diseases remains a difficult problem. To achieve this goal, it is important to develop new tools for effective infection screening and recognition. However, the lack of contrast at the boundary of lungs prevent the precise analysis of medical images. Many image processing and machine learning models have been developed for this purpose. In this work, the proposed framework is applied to identify which among the radiographic images are suspected or not of pneumonia.

**Figure 2:**
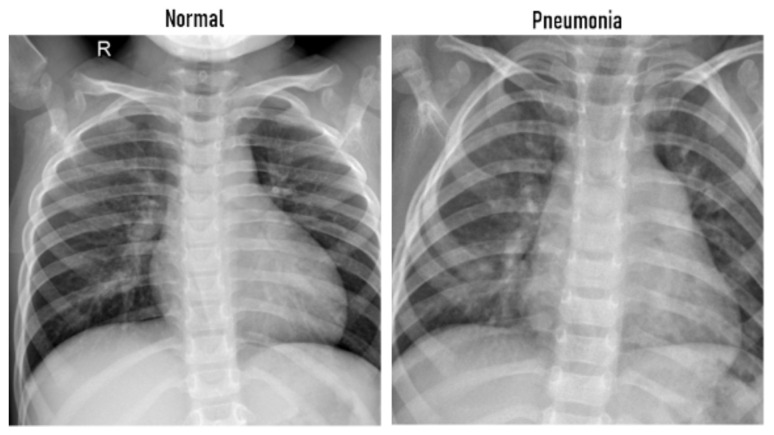
Examples of Chest X-Rays images. (**Left**) Normal patient, (**Right**) patient with pneumonia.

Our focus here is to evaluate the developed hybrid framework with some challenging pneumonia and COVID-19 datasets. The first application is related to COVID-19 detection. For this purpose, we considered the available dataset in [39,40] (https:https://github.com/ieee8023/covid-chestxray-dataset, accessed on 20 January 2021). This dataset contains 542 chest X-ray (CXR) images. A subset of 434 CXR images are positive for COVID-19 and the rest are normal cases. We also conduct our experiments to recognize pneumonia infections from CXR images. To achieve this purpose, we consider the publicly repository “Kaggle” (www.kaggle.com/paultimothymooney/chest-xray-pneumonia, accessed on 20 January 2021). It contains 5856 images divided into Pneumonia and Normal categories. There are 1583 normal images and 4273 pneumonia cases. An example of both normal and abnormal images from this dataset is given in Figure 2.

The proposed approach is built to discriminate between normal and abnormal lungs. First, a step of features extraction is computed using the Haralick feature [41,42]. The extraction step involves the calculation of the GLCM matrix [43]. In our experiments, we picked 70% of the dataset to be used for training purposes and the rest for testing and validation.

To evaluate the performance of our hybrid approach for lung diseases classification, we used the average accuracy (ACC), detection rate (DR), and false-positive rate (FPR) metrics. The accuracy (ACC) defines the overall well-classified images. The DR is the proportion of positive instances (vectors) in the data that are classified (or identified) correctly. The FPR is the proportion of negative instances that are classified incorrectly. A summary of the performance is displayed in Table 1 and Table 2. These tables show the obtained results using different methods based on Gaussian, Gamma, generalized Gamma, Dirichlet, scaled Dirichlet, and shifted scaled Dirichlet mixtures, respectively. In these experiments, we fed the SVM classifier with probabilistic kernels generated from different generative mixture models. According to these results, we can notice that our hybrid framework (shifted scaled Dirichlet mixture + SVM-kernels) achieves superior performance with ACC equal to 88.81% for the CXR-COVID dataset and 94.83% for the CXR-Pneumonia dataset. It also reaches a high detection rate (DR) and low false positive rate (FPR). It is clear that these values are considered very encouraging given that we approach the detection problem in an unsupervised manner. The Gaussian-based framework has the worst accuracy equal to 83.25% for CXR-COVID and 88.18% for CXR-Pneumonia. This degradation in performance confirms the fact that Gaussian mixtures are not flexible enough for complex multidimensional data. Furthermore, the accuracies increase as the size of the dataset increases (here, performance is better for the larger dataset: CXR-Pneumonia). According to these tables, when using shifted scaled Dirichlet mixture the average classification accuracy was better than the accuracy achieved by scaled Dirichlet using different kernels. Compared with Dirichlet and scaled Dirichlet, the lung disease recognition accuracy of shifted scaled Dirichlet was improved by 1% for CXR-COVID, and more than 1.5% for the CXR-Pneumonia dataset. This proves that the shifted scaled Dirichlet is a good choice and flexible enough to be applied in medical classification problems. We report that the developed framework does not take into account any background subtraction or pre-segmentation step and for this reason the accuracy is considered quite high. To improve more obtained findings, it may be a good idea to simultaneously incorporate a feature selection mechanism into the developed statistical discriminative framework.

### 5.2. Retinopathy Detection

**Figure 3:**
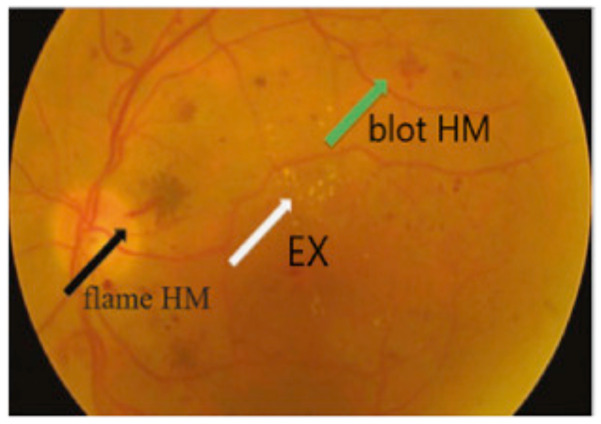
Types of hemorrhage (HM) [47].

Diabetic Retinopathy (DR) is a human eye disease and one of the most aggressive complications of diabetes, which causes damage to the retina. It is known that the augmentation of the quantity of glucose in the blood due to the lack of insulin leads to diabetes (https://www.idf.org/aboutdiabetes/what-is-diabetes.html, accessed on 20 January 2021). A previous study shows that diabetes affects the retina and nerves and can attack millions of people worldwide [44]. It has been found to be the principal leading cause of blindness among working elderly people worldwide, if not detected early [45]. It is reported by the International Diabetes Federation that Saudi Arabia and the Arab world have the largest amount of affected people (in 2014, 3.8 million diabetics in Saudi Arabia). Detection of diabetic retinopathy in early stages is one of the essential challenges and is very important to avoid complete blindness and for treatment success. Unfortunately, the accurate DR detection is time- and cost-consuming, known to be difficult and requires a trained specialist to identify the presence of lesions and abnormalities in digital retina photographs. Early and automated regular screening of DR is essential for good prognosis and can help speed up the process of decision-making and can notably reduce the risk of blindness of millions of people. Previous efforts have progressed well towards a comprehensive DR screening using image processing, data mining, and pattern recognition techniques. It is possible to detect DR by analyzing the presence of different types of lesions such as microaneurysms (MA), exudates (EX), and hemorrhages (HM) [46]. For instance, there are two types of HMs called blot (deeper HM) and flame (superficial HM). This lesion looks like larger spots on the image of the retina (see Figure 3). DR is usually distinguished into four stages: mild, moderate, severe, and proliferative. Generally, diseases begin with little changes in blood vessels (Mild DR) and it increases further to achieve severe and/or proliferative DR. At this final stage, if proper care is not taken, it will lead to blindness.

In the literature, various works have been carried out for retinal image classification and DR detection with interesting results. The study in [48] integrated a set of features of higher-order with SVM to classify eye images as DR or not-DR. A CNN model is developed in [49] for DR detection and macular edema (DME). The work in [50] introduced a method based on features extraction (AM-FM) to detect some kind of lesions and then try to measure their severity. Some works focus on classifying microaneurysms (MA) lesions by applying different methods like dynamic thresholding [51], wavelet transform [52], and a detector framework [53]. In [54], the authors proposed a method based on a convolution neural network to classify DR. Exudates lesions are detected in [55] by integrating fuzzy FCM and morphological operators into the same method. In [56], authors applied different machine learning algorithms (Decision Trees, SVM, ANN) to evaluate their performance in terms of DR prediction. Segmenting vasculature structures using robust segmentation techniques in retinal photographs may help in predicting DR at an early stage, as shown in [57], where an ensemble system and decision trees are investigated. The current problem of DR screening and classification is addressed in the present study. Indeed, we demonstrate the use of the developed hybrid generative discriminative framework for fundus images classification and DR detection.

The first step, even before applying our statistical model, is to extract relevant details (patterns) for retinal image classification. To address this step, we used “Speeded Up Robust Features (SURF)” extractor local features [58]. It is able to provide accurate description of input images. Each retinal image is then modeled through the developed shifted scaled Dirichlet mixture model. The last step is to classify the resulting descriptors on the basis of three constructed probabilistic kernels, which are deployed within an SVM classifier. These kernels are used to train the SVM. For this application, input observed vectors are divided into two subsets: training and testing subsets. We adopt a 10 fold cross-validation technique such that 70% of the vectors are considered during the training phase and the rest for testing. Our objective regarding the implementation of three different kernels is to evaluate their robustness and to analyze the stability of the SSDMM model.

It should be noted that we keep the same methodology for other models in order to conduct a correct comparative study. These models are Gaussian, Dirichlet, scaled and shifted scaled Dirichlet. The quantitative performance study is reported in terms of AUC (Area Under the Curve) and accuracy (ACC) metrics. Here AUC and ACC are performance measurements for the classification problem. They indicate how well the method is able to distinguish between patients with disease and those without disease (i.e., between different classes). Indeed, the higher the ACC and AUC, the better the method. In this study, we have considered two publicly available datasets named e-ophtha and DRIVE.

- E-ophtha [59]: this first dataset contains 47 images with EX and 35 normal images and includes 148 images with MA and 233 normal images.
- DRIVE [60]: This dataset includes 40 images with the size of 565 × 584 pixels where 7 are mild DR images, and the rest are normal retinal images.

The obtained results are given in Table 3 and Table 4. According to these results, it is clear that our discriminative framework based on the SSDMM model outperforms the other frameworks and gives the highest accuracy scores for both datasets. In particular, for the DRIVE dataset, the average accuracy of classifying retinal images using our method (SSDMM + Bhattacharyya kernel) is 91.65%, which is the best score. By contrast, the Gaussian-based classifier has obtained the worst score. For the second dataset (e-ophtha), we obtain the highest accuracy with our framework (SSDMM + Fisher kernel) with 96.88% compared to 96.07% for SDMM + Fisher kernel, 95.42% for DMM + Fisher kernel, and 94.84% for GMM + Fisher kernel. Here, the performance differences are statistically significant if we consider the Student’s t-test. Clearly, these results confirm the efficiency of our framework for modeling and classifying complex data. On the other side, the performance of the hybrid learning approaches outperform the generative mixture models (i.e., their counterparts) and also other methods from the literature for both datasets. It is noteworthy that the SSDMM mixture would be preferred here since it is more flexible than the rest of the models. It is also noted that when analyzing the reported values for the two datasets (DRIVE and e-ophtha), the accuracies of e-ophtha are better since its size is larger than DRIVE.

## 6. Conclusions

In this paper, we proposed an effective hybrid approach that considered the advantage of both generative and discriminative models. This approach utilizes a variant of a mixture model named the shifted-scaled Dirichlet mixture model (SSDMM), which is quite flexible to fit different shapes of observed data. In order to make our proposed framework particularly appropriate for image classification and abnormality detection problems, we derived new discriminative classifiers based on SVM kernels such as Fisher, Kullback–Leibler and Bhattacharyya for SSDMM. This strategy makes the developed framework more effective for complex and noisy data. Experiment results demonstrated that our approach has improved the accuracy when compared to other related methods in terms of accuracy over real CXR and retinal images. It has been found that our proposed algorithms outperformed many other methods. In particular, the highest accuracies obtained with our framework are 88.81% and 94.83% for CXR-COVID and CXR-Pneumonia datasets, respectively. On the other side, for the case of retinopathy detection, we achieved superior accuracies equal to 91.65% and 96.88% for both DRIVE and e-ophtha datasets, respectively. A potential future work could be devoted to extending the generative model to a non-parametric Bayesian principle in order to address the issue of accurate estimation of the number of components. We also plan to address other tasks such as image segmentation by classifying smaller regions in order to improve classification and categorization tasks.

## Figures and Tables

**Table 1:** Overall accuracy for the chest x-rays (CXR)-COVID dataset.

Approach/Metrics	ACC(%)	DR(%)	FPR(%)
**Generative Models**
Gaussian Mixture	82.11	81.02	0.18
Gamma Mixture	85.22	83.76	0.16
Dirichlet Mixture	87.80	85.92	0.13
Scaled Dirichlet Mixture	87.96	86.02	0.13
Shifted Scaled Dirichlet Mixture	88.01	86.12	0.12
**Hybrid Models**
Gaussian Mixture + Fisher Kernel	83.43	82.29	0.17
Gaussian Mixture + Kullback–Leibler Kernel	83.27	82.20	0.17
Gaussian Mixture + Bhattacharyya Kernel	83.25	82.18	0.17
Gamma Mixture + Fisher Kernel	86.01	84.11	0.16
Gamma Mixture + Kullback–Leibler Kernel	85.99	84.08	0.16
Gamma Mixture + Bhattacharyya Kernel	85.94	84.03	0.16
generalized Gamma Mixture + Fisher Kernel	87.01	87.90	0.12
generalized Gamma Mixture + Kullback–Leibler Kernel	87.71	87.01	0.12
generalized Gamma Mixture + Bhattacharyya Kernel	87.67	86.96	0.12
Dirichlet Mixture + Fisher Kernel	87.80	85.92	0.13
Scaled Dirichlet Mixture + Fisher Kernel	87.96	86.02	0.13
**Shifted Scaled Dirichlet Mixture + Fisher Kernel**	**88.81**	**86.91**	**0.11**
**Shifted Scaled Dirichlet Mixture + Kullback–Leibler Kernel**	**88.77**	**86.85**	**0.11**
**Shifted Scaled Dirichlet Mixture + Bhattacharyya Kernel**	**88.74**	**86.82**	**0.11**

**Table 2:** Overall accuracy for CXR-Pneumonia dataset.

Approach/Metrics	ACC(%)	DR(%)	FPR(%)
**Generative Models**
Gaussian Mixture	87.66	85.80	0.13
Gamma Mixture	90.54	88.54	0.10
Dirichlet Mixture	93.01	90.94	0.07
Scaled Dirichlet Mixture	93.33	91.90	0.07
Shifted Scaled Dirichlet Mixture	93.62	92.14	0.07
**Hybrid Models**
Gaussian Mixture + Fisher Kernel	88.25	86.90	0.12
Gaussian Mixture + Kullback–Leibler Kernel	88.22	86.83	0.12
Gaussian Mixture + Bhattacharyya Kernel	88.18	86.79	0.12
Gamma Mixture + Fisher Kernel	90.88	88.60	0.10
Gamma Mixture + Kullback–Leibler Kernel	90.85	88.53	0.10
Gamma Mixture + Bhattacharyya Kernel	90.84	88,51	0.10
generalized Gamma Mixture + Fisher Kernel	91.98	91.11	0.09
generalized Gamma Mixture + Kullback–Leibler Kernel	91.77	91.05	0.09
generalized Gamma Mixture + Bhattacharyya Kernel	91.75	91.02	0.09
Dirichlet Mixture + Fisher Kernel	93.01	90.94	0.07
Scaled Dirichlet Mixture + Fisher Kernel	93.33	91.90	0.07
**Shifted Scaled Dirichlet Mixture + Fisher Kernel**	**94.83**	**93.99**	**0.06**
**Shifted Scaled Dirichlet Mixture + Kullback–Leibler Kernel**	**94.51**	**93.82**	**0.06**
**Shifted Scaled Dirichlet Mixture + Bhattacharyya Kernel**	**94.48**	**93.77**	**0.06**

**Table 3:** Classification performance (%) comparison using different approaches for the DRIVE dataset.

Approach/Metrics	AUC	ACC
**Generative Models**
Gaussian Mixture	0.70	84.01
Dirichlet Mixture	0.72	84.79
Scaled Dirichlet Mixture	0.75	84.99
Shifted Scaled Dirichlet Mixture	0.77	85.36
**Hybrid Models**
Gaussian Mixture + Fisher Kernel	0.81	87.84
Gaussian Mixture + Bhattacharyya Kernel	0.81	89.02
Gaussian Mixture + Kullback–Leibler Kernel	0.81	87.11
Dirichlet Mixture + Fisher Kernel	0.84	88.54
Dirichlet Mixture + Bhattacharyya Kernel	0.86	90.67
Dirichlet Mixture + Kullback–Leibler Kernel	0.84	88.01
Scaled Dirichlet Mixture + Fisher Kernel	0.87	90.87
Scaled Dirichlet Mixture + Bhattacharyya Kernel	0.90	91.33
Scaled Dirichlet Mixture + Kullback–Leibler Kernel	0.85	88.14
Shifted Scaled Dirichlet Mixture + Fisher Kernel	0.88	91.13
Shifted Scaled Dirichlet Mixture + Bhattacharyya Kernel	0.91	91.65
Shifted Scaled Dirichlet Mixture + Kullback–Leibler Kernel	0.91	88.98
**Other Methods**
Fleming et al. [61]		89.80
Garcia et al. [62]		73.55
Li and Chutatape [63]		85.50
Wang et al. [64]		85.00

**Table 4:** Classification performance (%) comparison using different approaches for the e-ophtha dataset.

Approach/Metrics	AUC	ACC
**Generative Models**
Gaussian Mixture	0.81	81.45
Dirichlet Mixture	0.83	84.95
Scaled Dirichlet Mixture	0.83	85.34
Shifted Scaled Dirichlet Mixture	0.84	86.10
**Hybrid Models**
Gaussian Mixture + Fisher Kernel	0.90	94.84
Gaussian Mixture + Bhattacharyya Kernel	0.89	92.81
Gaussian Mixture + Kullback–Leibler Kernel	0.85	92.53
Dirichlet Mixture + Fisher Kernel	0.92	95.42
Dirichlet Mixture + Bhattacharyya Kernel	0.91	93.08
Dirichlet Mixture + Kullback–Leibler Kernel	0.88	93.77
Scaled Dirichlet Mixture + Fisher Kernel	0.95	96.07
Scaled Dirichlet Mixture + Bhattacharyya Kernel	0.94	95.91
Scaled Dirichlet Mixture + Kullback–Leibler Kernel	0.90	94.33
Shifted Scaled Dirichlet Mixture + Fisher Kernel	0.96	96.88
Shifted Scaled Dirichlet Mixture + Bhattacharyya Kernel	0.96	96.72
Shifted Scaled Dirichlet Mixture + Kullback–Leibler Kernel	0.93	95.12
**Other Methods**
linear-SVM [65]	0.89	85.33
RBF-SVM [65]	0.92	87.96
Random Forests [65]	0.92	95.08
Gaussian Processes [65]	0.93	87.62

## Data Availability

Data available in a publicly accessible repository: COVID-19 dataset available at: https://github.com/ieee8023/covid-chestxray-dataset, accessed on 20 January 2021; Pneumonia dataset available at: https://www.kaggle.com/paultimothymooney/chest-xray-pneumonia, accessed on 20 January 2021; E-ophtha dataset [59]; DRIVE dataset [60].
